# Macular micro vascular changes following macular hole repair: optical coherence tomography angiography study

**DOI:** 10.1186/s40942-026-00863-w

**Published:** 2026-05-26

**Authors:** Mohamed Hussein Mohamed Moustafa Hussein, Ahmed Tamer Sayed Seif, Mahrous Hassan Abd El Ghany Shaheen, Islam Abdallah Mohalhal

**Affiliations:** 1 Ophthalmology Department, Faculty of Medicine, Fayoum University, Fayoum, Egypt; 2 Retina Department, Research Institute of Ophthalmology, Giza, Egypt

**Keywords:** Macular microvascular, Macular hole repair, ILM peeling, ILM flap, Optical coherence tomography angiography, FAZ area

## Abstract

**Background:**

To detect changes in the macular vascular profile after macular hole closure using modified classic internal limiting membrane (ILM) peeling and temporal ILM flap techniques and their correlation with the macular microstructure and visual function.

**Patients and methods:**

Prospective comparative study that included 50 eyes with primary full-thickness macular hole (FTMH). Group A (*n* = 25) was treated using the modified classic ILM peeling technique, whereas group B (*n* = 25) was treated using the temporal ILM flap technique. The main outcome measures were macular hole closure, macular vascular and structural repair, and visual recovery rates. We performed OCT and OCTA using spectral-domain OCT (SD-OCT). The chi-square test was used to compare qualitative data, and the independent t-test was used to compare quantitative data. Analysis of variance test (ANOVA) was used to compare paired groups, and Spearman’s correlation coefficients were used to assess the correlations between quantitative parameters. P-value < 0.05.

**Results:**

Anatomical closure was comparable between groups A (80%) and B (88%) (*p* = 0.44). Both groups showed significant improvement in BCVA (*p* < 0.001), with no intergroup differences. The reduction in the FAZ area in the DCP was significant in both groups (*p* < 0.001). Only Group A showed a significant reduction in the FAZ in the SCP (*p* < 0.001). Preoperative hole dimensions correlated negatively with baseline BCVA and positively with FAZ in the SCP. Central macular thickness was inversely correlated with FAZ in both plexuses postoperatively. The magnitude of change in the FAZ in the DCP correlated negatively with the BCVA in groups A and B (*p* = 0.034 and *p* = 0.043, respectively).

**Conclusion:**

Free ILM peeling and temporal ILM flap techniques achieved comparable anatomical closure rates and sustained visual improvement. Preoperative hole dimensions are major determinants of structural remodeling and functional recovery, while OCTA reveals substantial microvascular rearrangement, especially within the DCP, which correlates with visual recovery.

**Clinical trial number:**

Not applicable.

**Supplementary Information:**

The online version contains supplementary material available at 10.1186/s40942-026-00863-w.

## Introduction

Macular hole (MH) is a full-thickness defect in the fovea, extending from the internal limiting membrane (ILM) to the retinal pigment epithelium (RPE). Macular holes cause profound vision loss, which manifests as a central scotoma. The etiology of the primary variant is multifactorial. It includes tangential traction applied to the fovea by contraction of the preretinal cortical vitreous and anteroposterior traction exerted by the posterior vitreous cortex on the neurosensory retina at the fovea [1].

Kelly and Wendel [2], in 1991, described vitrectomy procedure for MH closure. Improvements in surgical techniques, instrumentation, and visualization systems have promoted improvements in the anatomical and functional outcomes of MH surgery. The introduction of Internal Limiting Membrane (ILM) peeling by Eckardt et al. [3] significantly improved the success rates of surgery. More recently, modifications of ILM surgery have greatly enhanced the success rates, particularly in large and refractory macular holes [1, 4, 5].

The introduction of ultra-high-speed optical coherence tomography (UHR-OCT) has provided insights into the vitreoretinal interface and ultrastructure of the retinal layers in eyes with MH [6, 7]. It helped understanding the pathogenetic mechanisms underlying poor visual outcomes in patients with successful MH closure after surgery [1]. UHR-OCT showed that recovery of the external limiting membrane, photoreceptor inner and outer segment junction line, and cone outer segment tip line (COST) were associated with functional recovery after MH surgery [8].

Integrated OCT angiography (OCTA) technology enables noninvasive imaging of the vascular layers of the retina and choroid [9, 10]. Moreover, it provided evidence that the foveal avascular zone (FAZ) area is correlated with central macular thickness (CMT) and visual acuity in healthy normal population [11]. Additionally, prior research indicated that OCTA assessments conducted postoperatively after MH closure demonstrated marked alterations in the architecture of the macular capillary plexus [12].

The aim of this study is to detect the changes in the vascular profile of the macular area in patients with successful closure of macular holes after pars plana vitrectomy (PPV) using either a modification of the classic ILM peeling technique described by Eckardt et al. (1997) [3] or the temporal ILM flap technique [13] and to correlate these changes with microstructural changes in the retinal layers and visual function.

## Patients and methods

This prospective comparative consecutive interventional quasi-randomized study compared the modified classic ILM peeling and temporal ILM flap techniques in patients with primary full-thickness macular holes (FTMH). The study was conducted at two retinal tertiary centers in Egypt between 2021 and 2025. The patients were divided into two groups. The recruited patients were randomly assigned to groups A and B alternatingly according to the order of their attendance. This allocation was performed by the vitreoretinal resident in the clinic (who was completely blinded to the study) once the FTMH diagnosis was confirmed, before presenting the case to the surgeon. Patients in group A received the modified classic ILM peeling technique, and patients in group B received the temporal ILM flap technique. The study exclusively included patients with treatment-naïve primary MH. The exclusion criteria were associated retinal detachment; patients with previous PPV; high myopia with axial length ≥ 26 mm; uncertain symptom duration or duration ≥ 6 months; full-thickness macular holes secondary to trauma; associated retinal pathology such as retinal vascular diseases; and significant media opacities that could impede visualization or result in poor image quality.

The main outcome measures were macular hole closure, macular vascular and structural repair, and visual recovery. All recruited patients underwent a full preoperative assessment, including history taking, best corrected visual acuity (BCVA) using a Snellen chart (which was later converted to the logarithm of the minimal angle of resolution; logMAR for statistical analysis), slit-lamp examination of the anterior segment, posterior segment examination using a binocular indirect ophthalmoscope, and slit-lamp biomicroscopy using a + 78 dpt (D) Volk lens for detailed evaluation of the macula, axial length measurement, spectral domain OCT (SD-OCT), and OCTA imaging (Spectralis tracking laser tomography, Heidelberg Engineering GmbH, Heidelberg, Germany).

**Fig. 1:**
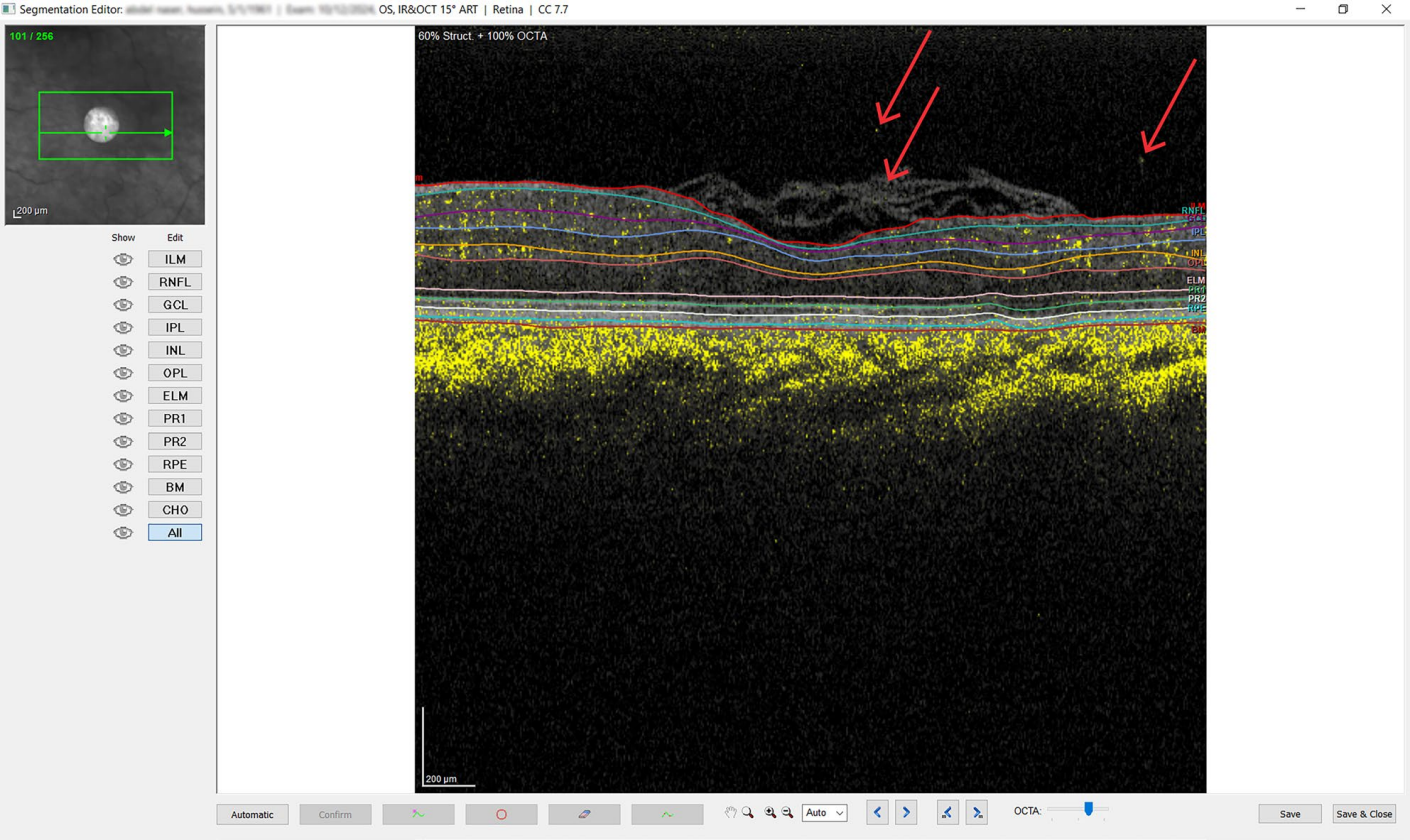
Presents a screenshot from the OCTA program, demonstrating the segmentation editor software employed by the investigator to detect and rectify segmentation errors. The case presented serves as an illustration of a manual adjustment of the ILM (indicated by the red line) at the upper surface of the retina, effectively excluding the previously erroneously included folded ILM flap and associated artifacts (yellow dots highlighted by red arrows)

The SD-OCT scanning protocol included a radial scan consisting of a sequence of six radial sections centered on the macular hole. The radial sections were spaced by 30⸰ and covered the central 15°. The scan was recorded in high-resolution mode (768 A-scans). We defined the minimum linear diameter, (MLD) as the narrowest width of the hole at its inner opening, the basal linear diameter (BLD) as the widest measurement of the hole at the level of the retinal pigment epithelium (RPE), and the hole height (HH) as the vertical distance between the junction of the attached and detached photoreceptor layer and the ILM. OCTA scans of the superficial vascular plexus (SCP) and deep vascular plexus (DCP) were captured by a sequence of (256–512) sections covering the central (10° × 5°–10° ×10°) recorded in the high-resolution mode and spaced by 6 μm. SCP and DCP were distinctly evaluated using the automatic layer segmentation feature available in the software. Furthermore, the investigator checked all the sections to ensure that accurate automatic segmentation was done, and in cases of segmentation errors or artifacts, the investigator used various tools of the “segmentation editor” incorporated in the software to manually correct the false segmentation (Fig. 1). The SCP was segmented from the ILM to the inner nuclear layer (INL), and the DCP was segmented from the INL to the outer plexiform layer (OPL). We defined the FAZ as the inner border of the most visible central blood capillaries. We used the software “Draw region” tool to manually outline the area of the FAZ in both layers, and the software automatically calculated the outlined area. The manual adjustments and measurements were performed by an independent investigator who was blinded to all other variables.

### Surgical procedure

**Fig. 2:**
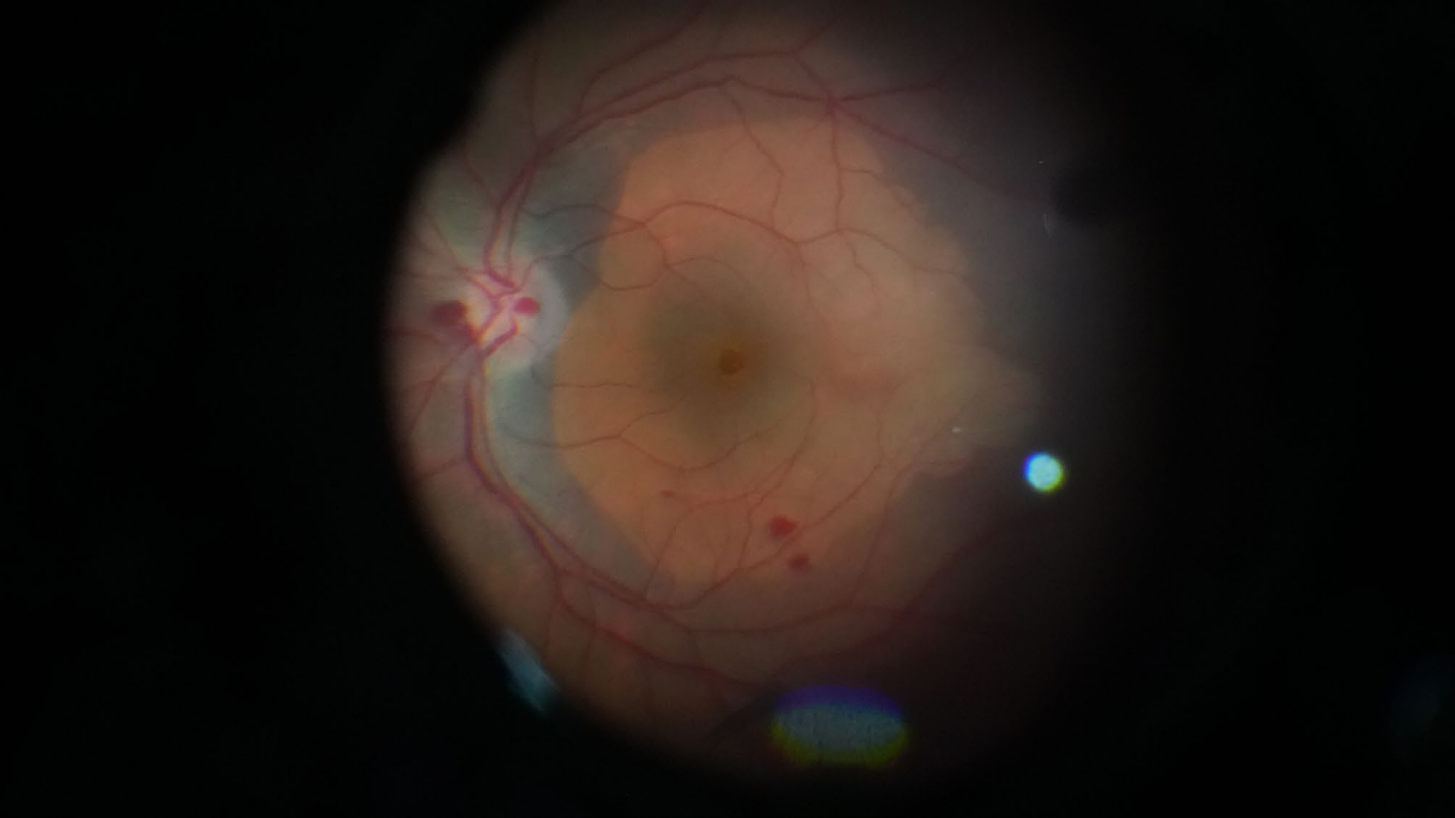
Surgical photograph of free ILM peeling

**Fig. 3:**
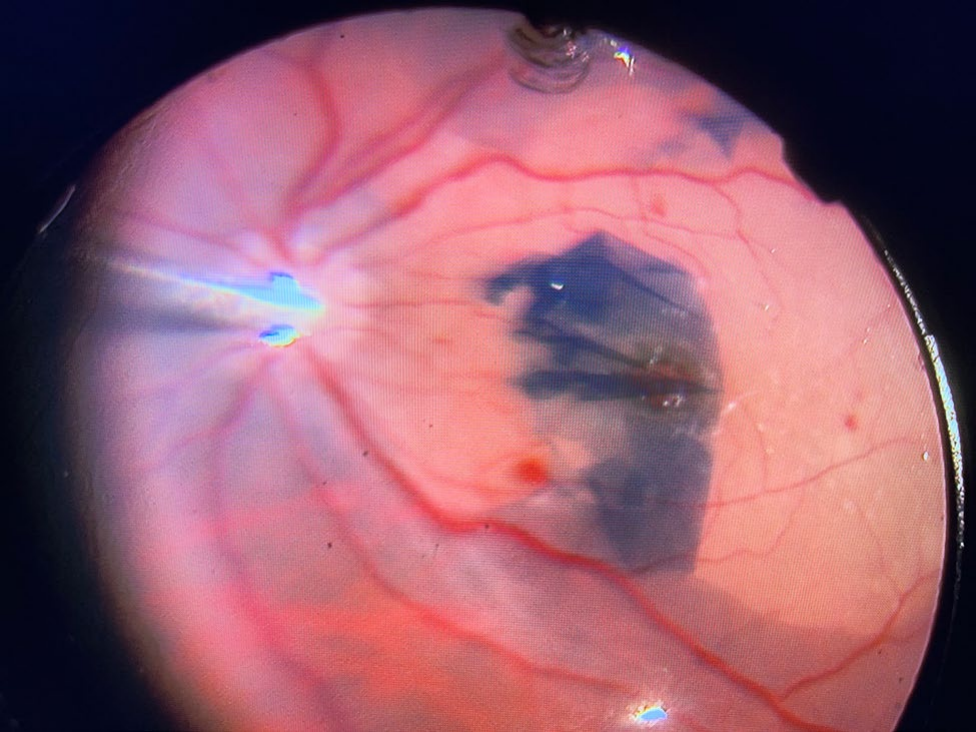
Surgical photograph of ILM temporal Flap adequately covering the macular hole

The surgical procedure consisted of 23-gauge PPV, which included posterior vitreous detachment induction aided by triamcinolone acetonide, core vitrectomy, and minimal vitreous base shaving. The surgeon then proceeded to ILM staining using 0.025% brilliant blue G stain (Dutch Ophthalmic Research Center (DORC), The Netherlands). After aspirating the residual stain, ILM peeling was initiated using Tano’s diamond-dusted scraper or by the direct pinching technique using vitreoretinal forceps. In group A, the surgeon used a modification of the classic ILM peeling technique that consisted of a maculorrhexis to peel the ILM in a circular pattern extending from arcade to arcade, and approximately 2–3 disc diameters temporal to the fovea. (Fig. 2). Additional file 1: Videos S1, supplemental digital content (1) In group B, the surgeon used the temporal ILM flap technique, which consisted of detaching the ILM two-disc diameters away from the temporal aspect of the macular hole. During this procedure, the ILM remained partially attached to the retina at the temporal edge of the macular hole, and was subsequently inverted and gently positioned over the hole to ensure sufficient coverage. (Fig. 3). Additional file 2: Videos S2, supplemental digital content (2) The flap is stabilized by fluid-air exchange while gently aspirating and using the fluid currents to spread the flap over the hole. In both groups, the surgeon used air tamponade. We advised all patients to adopt a face-down position for five days. A single vitreoretinal surgeon (MHM) performed all surgical procedures.

We performed regular assessments at 1, 3-, and 6-months postoperatively. The assessment included BCVA measurement, slit-lamp biomicroscopy, fundus examination, and OCT and OCTA imaging. We used the aforementioned OCT and OCTA protocols for postoperative imaging of the macula. In addition, we measured the macular thickness map to measure the central macular thickness (CMT). The SD-OCT thickness map protocol consisted of a sequence of 19 horizontal sections covering the central 15° recorded in the high-resolution mode and spaced by approximately 240 μm between individual sections. CMT was measured automatically as the central 1 mm sub-field thickness of the early treatment diabetic retinopathy study (ETDRS) layout. In addition, we measured the paracentral 3 mm of the ETDRS layout in the nasal and temporal quadrants. The software automatically calculated these measurements.

### Statistical analysis

The sample size of the study was 50 eyes; this determination was grounded on the ELM recovery rate observed in ILM peeling, which was 70%, in contrast to the 21.4% recovery rate associated with inverted flaps [14]. Furthermore, with a statistical power of 80% and a confidence level of 95%, the sample size has been computed utilizing openEpi software, yielding a requisite of 16 eyes in each group.

Data were collected, revised, coded and entered into the Statistical Package for Social Science (IBM SPSS) version 23. Quantitative data were presented as mean, standard deviation, and range when parametric and median. Qualitative variables were presented as numbers and percentages.

Qualitative data were compared between groups using the chi-square test. The comparison between two independent groups with quantitative data and parametric distribution was done using the independent t-test. The comparison between more than two paired groups regarding quantitative data and parametric distribution was done by using Repeated Measures ANOVA test with post hoc by Bonferroni test. Spearman correlation coefficients were used to assess the correlations between the two quantitative parameters in the same group.

The confidence interval was set to 95%, and the margin of error accepted was set to 5%. So, the p-value was considered significant as follows: p-value > 0.05: non-significant (NS); p-value < 0.05: significant (S); and p-value < 0.01: highly significant (HS).

## Results

The study included 50 eyes of 48 patients. We divided the patients evenly into two groups, A and B, each containing 25 patients. Twenty-seven patients were female (54%). The mean age was 64 years (range: 49–78, SD 10). Thirty-one patients (62%) had MH in their right eye. No statistically significant differences were found between groups A and B regarding the demographic and baseline parameters, except for the BHD, which was significantly larger in group B (*p* = 0.026; Table 1).

**Table 1:** Comparison between group A and group B regarding demographic data and baseline parameters of the MH

		Group A	Group B	*P*-value
		no. = 25	no. = 25	
Age (years)	Mean	66	62	0.284
	Range	50–75	49–78	
Gender	Female	16	11	0.156
	Male	9	14	
Eyes	OS	15	16	0.771
	OD	10	9	
BCVA (LogMar)	Mean	1.21	1.24	0.387
Hole size /µm				
BHD	Mean	1011	1240	0.026*
MLD	Mean	504	596	0.090
HH	Mean	394	409	0.589
SCP FAZ/mm2	Mean	0.60	0.62	0.738
DCP FAZ/mm2	Mean	0.70	0.65	0.480

BCVA best corrected visual acuity; BHD basal hole diameter; MLD minimum linear diameter; HH hole height; SCP superficial capillary plexus; FAZ fovea avascular zone; DCP deep capillary plexus

Statistical analysis revealed that in both groups, BHD and MLD were negatively correlated with preoperative visual acuity (positively correlated with logMAR BCVA; *p* = 0.02 and *p* = 0.000, respectively, in group A, and *p* = 0.01 and *p* = 0.001, respectively, in group B). Moreover, the BHD and MLD correlated positively with the FAZ area in the SCP layer (*p* = 0.009 and *p* = 0.043, respectively, in group A, and *p* = 0.01 and *p* = 0.000, respectively, in group B).

### Anatomical outcome

**Fig. 4:**
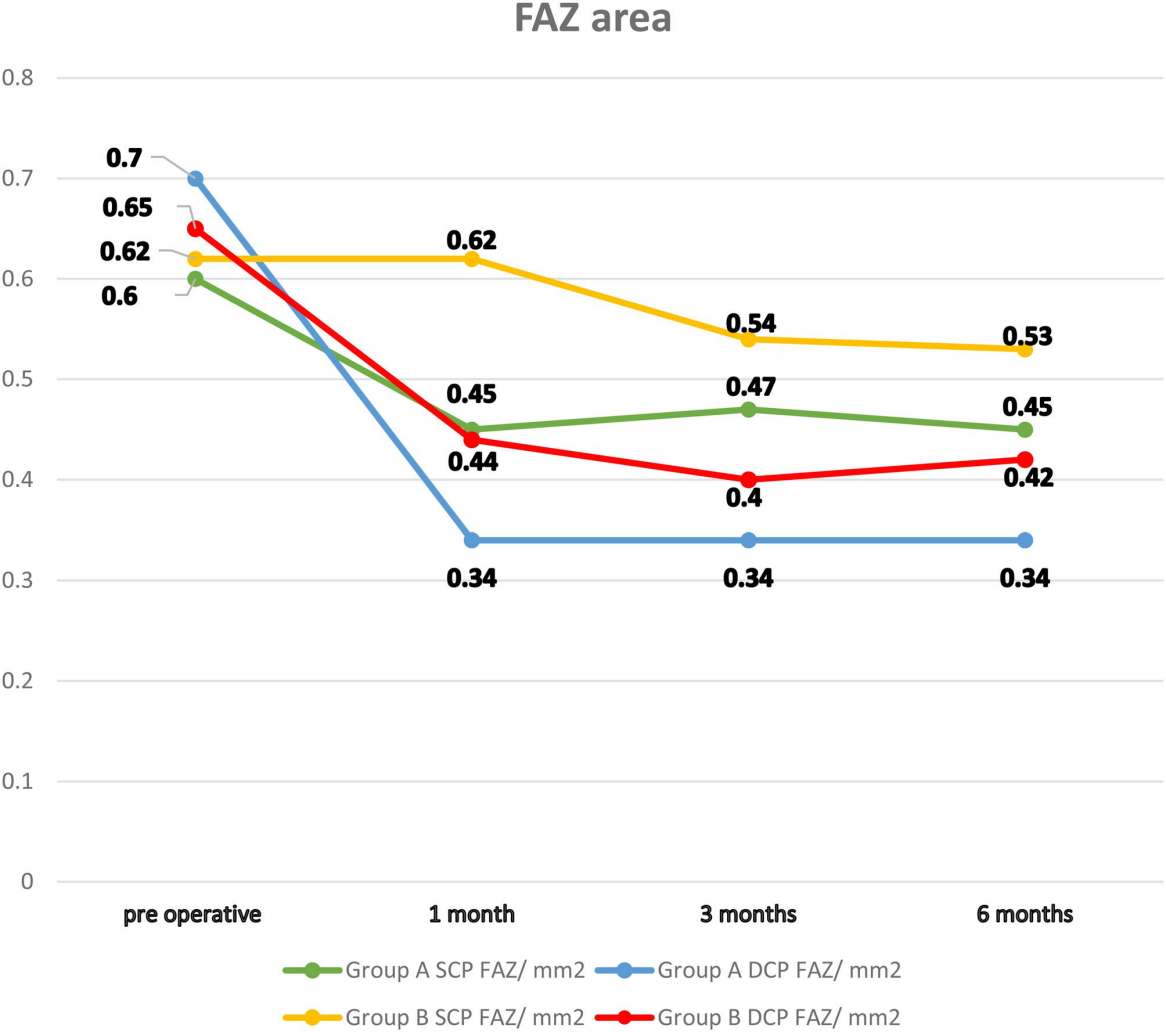
Presents the longitudinal assessment of the FAZ area in both the SCP and DCP across both groups, evaluated preoperatively and at the 1-, 3-, and 6-months postoperative intervals. FAZ fovea avascular zone; SCP superficial capillary plexus; DCP deep capillary plexus

**Fig. 5:**
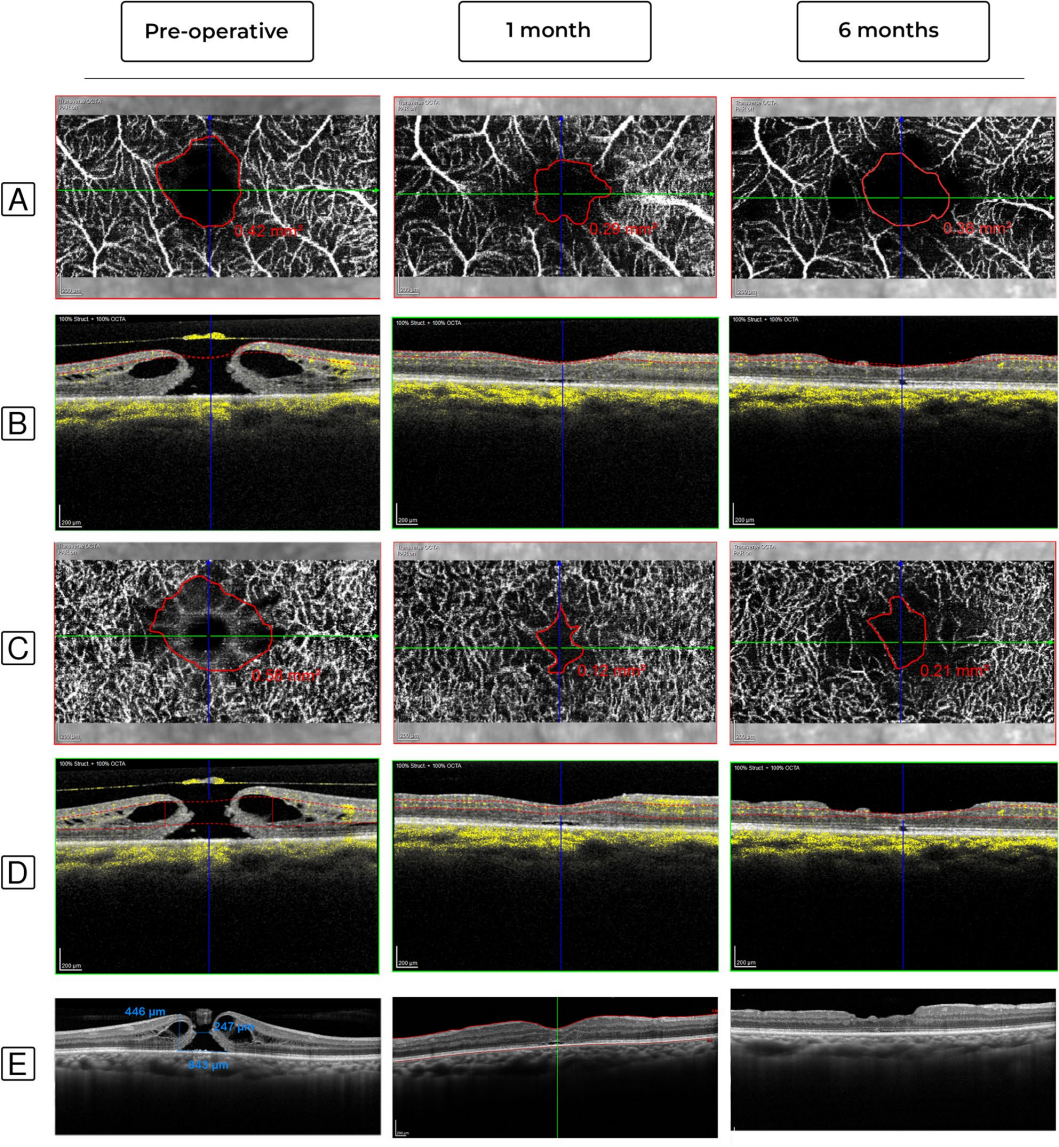
Represents a case that underwent modified classic ILM peeling without a flap, with significant FAZ area reduction. **(A–D)** En face OCTA and B-scan images at the preoperative, 1- and 6-months follow-up appointments. **(A-B)** FAZ area at the SCP changed from 0.42 mm2 preoperatively to 0.29 mm2 at the 1-month follow-up, and then to 0.38 mm2 at the 6-months follow-up. **(C-D)** FAZ area at the DCP changed from 0.58 mm2 preoperatively to 0.12 mm2 at the 1-month follow-up, and then to 0.21 mm2 at the 6-months follow-up. **(E)** B-scan images of the fovea at the preoperative, 1- and 6-months follow-up visits. The preoperative MLD, BHD and HH were 247, 843, and 446 μm, respectively. At the 1-month follow-up, the hole was successfully closed with partial ELM recovery. At the 6-months follow-up, the ELM was almost completely restored

**Fig. 6:**
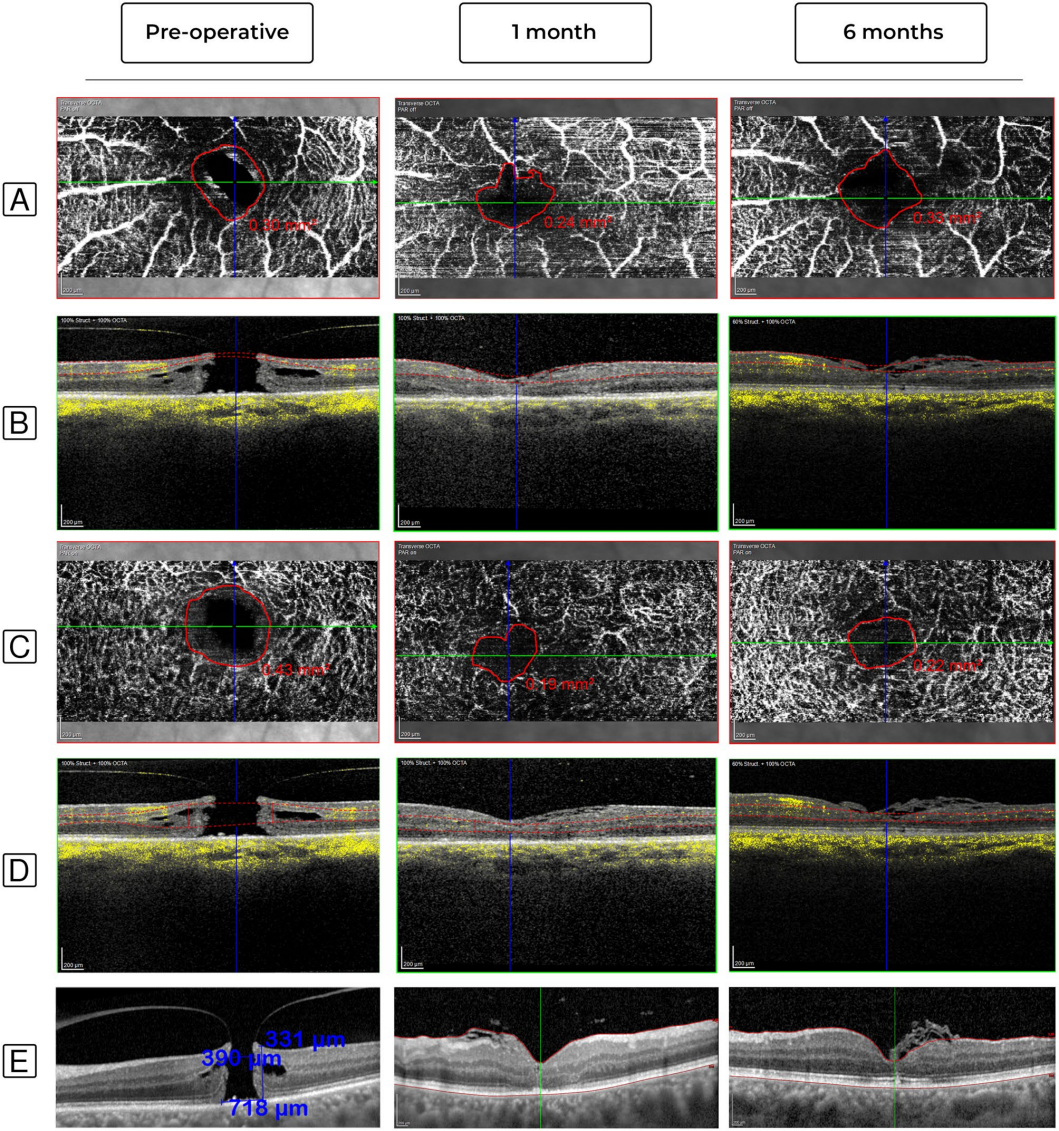
Represents a case that underwent ILM peeling with the temporal flap technique, with significant FAZ area reduction at the DCP and an almost stable FAZ area at the SCP. **(A–D)** En face OCTA and B-scan images at the preoperative, 1- and 6-months follow-up appointments. **(A-B)** FAZ area at the SCP changed from 0.30 mm2 preoperatively to 0.24 mm2 at the 1-month follow-up, and then to 0.33 mm2 at the 6-months follow-up. **(C-D)** FAZ area at the DCP changed from 0.43 mm2 preoperatively to 0.19 mm2 at the 1-month follow-up, and then to 0.22 mm2 at the 6-months follow-up. **(E)** B-scan images of the fovea at the preoperative, 1- and 6-months follow-up visits. The preoperative MLD, BHD and HH were 390, 718, and 331 μm, respectively. At the 1-month follow-up, the hole was successfully closed with partial ELM recovery. At the 6-months follow-up, the ELM was almost completely restored

Statistical analysis revealed comparable closure rates in both groups. Group A, 80% and group B, 88%, (*p* = 0.44). In group A, four eyes had W-shaped closure, and one eye developed CNV after successful closure (five eyes from group A were excluded from the study). In contrast, in group B, two eyes had a dislocated ILM flap, and one eye had a lost flap (three eyes from group B were excluded). The eight patients underwent different interventions and were excluded from the study prior to any postoperative statistical calculations. Postoperatively, in group A, there was a statistically significant reduction in the area of the FAZ in the SCP layer compared to the baseline (*p* < 0.001) and in the DCP layer in both groups (*p* < 0.001). Bonferroni post-hoc analysis revealed that the reduced FAZ area in both vascular plexuses remained stable after the first month postoperatively (Figs. 4, 5 and 6).

The comparison between the mean values of the different postoperative study parameters in groups A and B throughout the follow-up visits revealed that only the FAZ area in the SCP layer in group A was significantly smaller than that in group B in the first postoperative month. In group A, the mean FAZ area in the SCP layer was 0.449 mm2, and in group B, the mean value was 0.623 mm2 (*p* = 0.026; Table 2).

**Table 2:** Comparison between group A and group B regarding 1-month follow up data

1-month follow up		Group A	Group B	Test value (t)	*P*-value
		no. = 20	no. = 22		
BCVA	Mean ± SD	0.93 ± 0.18	0.93 ± 0.17	-0.041	0.967
SCP FAZ /mm2	Mean ± SD	0.449 ± 0.18	0.623 ± 0.29	-2.311	0.026*
DCP FAZ/mm2	Mean ± SD	0.34 ± 0.17	0.44 ± 0.18	-1.962	0.057
CMT/µm	Mean ± SD	258.05 ± 61.07	254.95 ± 76.58	0.144	0.886

BCVA best corrected visual acuity; SCP superficial capillary plexus; FAZ fovea avascular zone; DCP deep capillary plexus; CMT central macular thickness

### Functional outcome

**Fig. 7:**
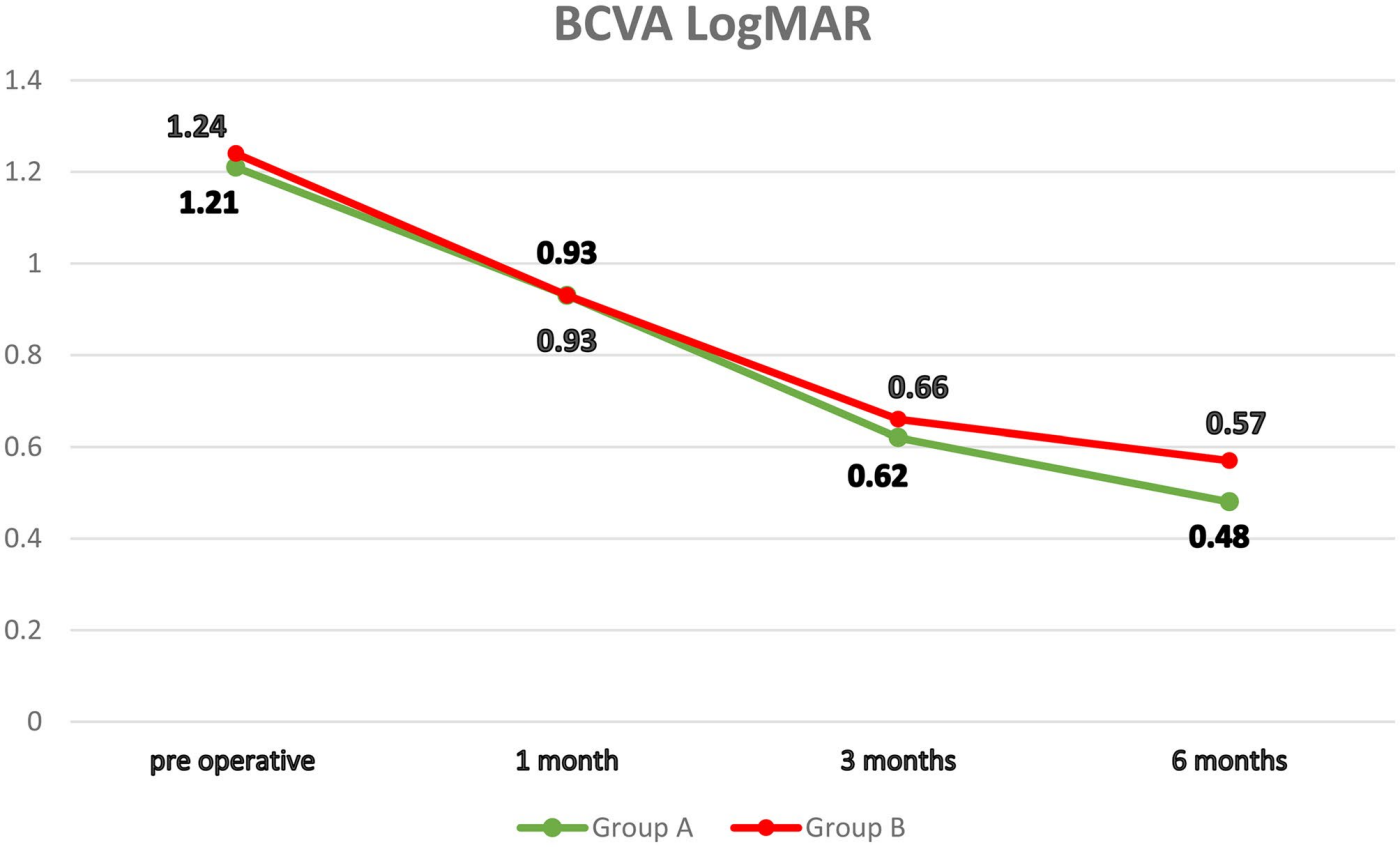
The follow-up of BCVA measured in LogMAR was conducted at the pre-operative stage, as well as at the 1-month, 3-month, and 6-month follow-up appointments for groups A and B. BCVA best corrected visual acuity

Statistical analysis in both groups showed a significantly progressive improvement in BCVA across the three follow-up visits compared to the baseline values. The final mean BCVA improved by more than five lines of vision in both groups (*p* < 0.001; Fig. 7).

### Statistical correlation

In group A, MLD had an inversely proportional correlation with postoperative visual acuity at the end of the follow-up period (directly proportional to LogMAR BCVA; *p* = 0.001). Moreover, at the end of the follow-up period, HH had a directly proportional correlation with CMT (*p* = 0.006) and an inversely proportional correlation with the FAZ area in the SCP layer (*p* = 0.045). The FAZ area in the DCP layer had an inversely proportional correlation with visual acuity in the first month postoperatively only (directly proportional to LogMAR BCVA; *p* = 0.026). Meanwhile, CMT was inversely proportional with the FAZ area in the SCP and DCP layers at the end of the follow-up period (*p* = 0.001 and *p* = 0.026, respectively; Table 3).

**Table 3:** Correlation of BCVA and CMT with FAZ area at the three Follow ups in Both groups

**Group A**				
**1-month follow up**	**BCVA (LogMar)**		**CMT/µm**	
	**r**	**P-value**	**r**	**P-value**
SCP FAZ /mm2	0.391	0.088	**-0.701** ^**^	**0.001**
DCP FAZ/mm2	0.496*	**0.026**	**-0.501** ^*^	**0.025**
**3-months follow up**	**BCVA (LogMar)**		**CMT/µm**	
	**r**	**P-value**	**r**	**P-value**
SCP FAZ /mm2	0.401	0.079	**-0.538** ^*^	**0.014**
DCP FAZ/mm2	0.169	0.475	-0.422	0.064
**6-months follow up**	**BCVA (LogMar)**		**CMT/µm**	
	**r**	**P-value**	**r**	**P-value**
SCP FAZ /mm2	0.096	0.688	**-0.667** ^**^	**0.001**
DCP FAZ/mm2	0.131	0.581	**-0.497** ^*^	**0.026**
**Group B**				
**1-month follow up**	**BCVA (LogMar)**		**CMT/µm**	
	**r**	**P-value**	**r**	**P-value**
SCP FAZ /mm2	-0.005	0.981	**-0.685** ^**^	**0.000**
DCP FAZ/mm2	0.285	0.198	-0.388	0.075
**3-months follow up**	**BCVA (LogMar)**		**CMT/µm**	
	**r**	**P-value**	**r**	**P-value**
SCP FAZ /mm2	0.245	0.272	**-0.774** ^**^	**0.000**
DCP FAZ/mm2	0.070	0.756	**-0.644** ^**^	**0.001**
**6-months follow up**	**BCVA (LogMar)**		**CMT/µm**	
	**r**	**P-value**	**r**	**P-value**
SCP FAZ /mm2	0.308	0.164	**-0.598** ^**^	**0.003**
DCP FAZ/mm2	**0.554** ^**^	**0.007**	**-0.659** ^**^	**0.001**

BCVA best corrected visual acuity; CMT central macular thickness; SCP superficial capillary plexus; FAZ fovea avascular zone; DCP deep capillary plexus

In group B, HH showed a direct correlation with CMT at the end of the follow-up period (*p* = 0.011). CMT was inversely proportional to the FAZ area of both the SCP and DCP at the end of the follow-up period (*p* = 0.003 and *p* = 0.001, respectively; Table 3). In addition, the FAZ area in the DCP layer had an inversely proportional correlation with visual acuity at the end of the follow-up period (directly proportional to LogMAR BCVA; *p* = 0.007; Table 3).

There was a significant negative correlation between the magnitude of the change in the FAZ area in the DCP over the study period and visual acuity in both groups (positive correlation with LogMAR BCVA; *p* = 0.034 and *p* = 0.043 in groups A and B, respectively).

## Discussion

This prospective comparative study provides comprehensive insights into the anatomical, microvascular, and functional outcomes of idiopathic FTMH surgery using two distinct surgical approaches. Our findings demonstrate that both surgical techniques achieve comparable anatomical closure rates and result in significant visual improvements. Notably, OCTA revealed substantial microvascular remodeling in both groups, with a significant reduction in the FAZ area, predominantly affecting the DCP layer.

Ramtohul et al. [15], reported closure rates of 70% with complete ILM peeling and 96% with inverted ILM flap technique in large macular holes greater than 400 μm (*p* = 0.02). Conversely, our closure rates were 80% and 88% using the classic ILM peel and temporal ILM flap techniques, respectively. The higher success rate in our study using complete ILM peel could be attributed to the inclusion of a wider range of hole sizes, where 30% of our patients had smaller holes < 400 μm. Michalewska et al. [13] first described the inverted ILM flap technique for large macular holes, achieving 98% closure rate and emphasizing that flap displacement remains a potential complication requiring meticulous surgical technique. In agreement with Michalewska et al. [13], we found that flap displacement was the predominant cause of failed hole closure.

Importantly, preoperative hole dimensions were critical predictors of both structural remodeling and functional recovery. The preoperative correlation analysis revealed that both BHD and MLD were negatively correlated with the baseline BCVA and positively correlated with the baseline FAZ area in the SCP layer in both groups. These findings are consistent with those of Ramtohul et al. [15] and Kim et al. [12], who reported that larger macular hole dimensions correlated with worse preoperative best-corrected visual acuity. They postulated that larger hole dimensions are associated with greater photoreceptor disruption and foveal tissue loss, more extensive retinal structural damage, and reduced foveal sensitivity.

Our findings regarding the positive correlation between BHD and MLD and the FAZ area in the SCP layer are congruous with those of Cho et al. [16], who documented comparable findings in their OCTA study of surgically closed macular holes. The authors proposed that larger holes trigger peripheral vascular recruitment mechanisms as compensatory vascular remodeling, potentially representing an adaptive response to central capillary loss.

Our study found that preoperatively, the FAZ areas in the SCP and DCP layers showed no significant difference between the two groups. However, postoperative patterns diverged notably between the surgical techniques. Although significant narrowing of the FAZ area in the DCP layer persisted throughout the follow-up period in both groups, in group A only, the FAZ area in the SCP layer decreased significantly from the baseline measurement to the first-month follow-up and then remained stable during subsequent assessments. These findings are consistent with those of Tsuboi et al. [17], who mentioned that the acute surgical effect associated with successful closure of the hole is followed by a phase of stabilization due to excessive centripetal movement of the retinal tissue. Conversely, group B maintained a relatively stable FAZ area in the SCP layer throughout the study without a statistically significant overall change compared to baseline values.

In addition, at the first-month follow-up, group B demonstrated a significantly larger FAZ area in the SCP layer than group A. These differential vascular responses between the techniques represent one of the key findings of our study. Similarly, previous studies have reported greater retinal vascular remodeling in areas where the ILM was peeled than in those where the ILM was preserved [18, 19]. However, this varied vascular response did not correlate with functional recovery, indicating no impact on visual restoration.

The comparable final visual outcomes between the two groups, despite persistent vascular differences, indicate robust functional compensation mechanisms, as proposed by Yun et al. [6], who suggested that compensation involves enhanced reliance on the remaining vascular supply and metabolic adaptation. The authors demonstrated that, despite asymmetric vascular remodeling, functional recovery correlated primarily with structural restoration rather than with vascular density parameters.

Our results are in agreement with those of Kim et al. [12], who investigated macular capillary plexuses after macular hole surgery using OCTA in 33 patients followed for ≥ 6 months, reporting that eyes after MH surgery had smaller FAZ areas in both the superficial and deep capillary plexuses (*p* < 0.05 for all) and reduced parafoveal vascular density compared to fellow eyes. However, their study did not differentiate between surgical techniques and examined only eyes that underwent standard vitrectomy with ILM peeling.

Both groups demonstrated highly significant progressive improvement in BCVA from baseline through all follow-up visits, with continued visual gains between all consecutive time points. However, no statistically significant difference in BCVA was found between the groups at any follow-up point, indicating comparable functional outcomes despite the different surgical techniques. Ramtohul et al. [15] similarly found no significant difference in final visual acuity between inverted ILM flap technique and complete ILM peeling groups despite the flap technique achieving higher anatomical closure rates, suggesting that when successful closure is achieved, both techniques provide comparable functional outcomes.

Correlation analysis in group A revealed significant negative relationships between preoperative horizontal hole dimensions and postoperative BCVA at all follow-up points. These findings align with those of Ramtohul et al. [15], who demonstrated that a larger MLD was significantly associated with worse postoperative BCVA (*p* = 0.03), with holes > 400 μm showing poorer visual outcomes than smaller defects. Kim et al. [12] reported similar negative correlations proposing that larger holes cause more extensive photoreceptor damage and greater retinal tissue displacement, limiting recovery potential. This finding is in line with that of Michalewska et al. [13], who emphasized in their landmark study that larger macular holes (> 400 μm) present greater surgical challenges and demonstrate poorer visual recovery despite successful anatomical closure. In addition, Itoh et al. [20] demonstrated that smaller holes showed more complete and symmetric restoration of the ellipsoid zone and external limiting membrane, which are the primary determinants of functional recovery. These observations advocate for early surgical intervention before extensive hole enlargement [21].

Our study found that in group A, holes with greater HH showed a greater SCP FAZ area reduction and increased central retinal thickness during postoperative recovery, potentially reflecting more extensive tissue rearrangement or glial proliferation during healing. This observation aligns with the findings of Kim et al. [22], who investigated the asymmetric elongation of foveal tissue after macular hole surgery and demonstrated that vertical hole dimensions influence tissue distribution patterns during healing. This may reflect that holes with larger HH involve greater tissue elevation without necessarily wider photoreceptor loss, leading to better postoperative restoration, or alternatively, that vertical configuration influences glial cell bridging patterns during closure. Wilczyński et al. [23] investigated OCTA features in patients with idiopathic full-thickness macular holes before and after surgical treatment, reporting that macular hole closure was associated with significant changes in central retinal thickness and that the healing process involved complex structural remodeling extending beyond simple hole closure.

SCP and DCP FAZ areas were negatively correlated with CMT in both groups throughout the study. These findings align with those of Tsuboi et al. [17], who reported that a smaller FAZ area at the beginning of recovery postoperatively, along with a thickened central retina, can be justified by the excessive centripetal migration of the retinal tissue. Although the exact MH closure process is not yet agreed upon, one possible mechanism is the glial proliferation of the inner retina bridging the hole to close it, leading to centripetal tissue movement, which might cause thickening of the central retina and narrowing of the FAZ area early in the postoperative period. This complex relationship between perfusion and anatomical restoration suggests that retinal tissue healing potential relies on vascular and neuronal plasticity, proposing that recovery after MH surgery may be involved in both anatomic and hemodynamic changes in the retina. On the other hand, Wilczyński et al. [23], who compared the results of a single follow-up point three months after successful closure with the preoperative values, found a mean CMT reduction from 396.75 ± 62.6 to 272.17 ± 30.7 μm postoperatively (*P* = 0.001). While aligning with our findings, they found that surgically closed macular holes demonstrated a reduction in FAZ from 0.39 ± 0.07 mm² preoperatively to 0.24 ± 0.07 mm² postoperatively (*P* < 0.001). Wilczyński et al. [23] attributed this to the presence of cystic changes in the middle retina on en face scans associated with MH which was resolved after closure, likely resulting in contraction of the FAZ with blood vessels and retinal tissue replacing the fluid-filled cystic areas with subsequent reduction of CRT. Although the same cystic changes were observed in our study, we did not measure preoperative CMT as Wilczyński et al. [23] did.

Our study findings indicate that Foveal Avascular Zone (FAZ) area measurements did not correlate with visual acuity across all follow-up points. These results align with those of Yun et al. [24] and Wilczyński et al. [23], who observed that retinal vascular alterations often do not correspond to visual outcomes. Tsuboi et al. [17] proposed that this lack of correlation may stem from individual variability in baseline FAZ dimensions, complicating its utility as a reliable biomarker. Conversely, the change in the FAZ area appears more significant; our data revealed a negative correlation between postoperative FAZ shifts and visual acuity. Similar observations were made by Tsuboi et al. [17] suggests that the expansion of the FAZ toward normal parameters following an initial postoperative contraction serves as a marker for successful foveal reconstruction after Macular Hole (MH) closure. This illustrates that foveal recovery involves a comprehensive reconstruction process encompassing both photoreceptor repair and restoration of the standard foveal architecture (such as Central Retinal Thickness and FAZ area). In contrast, Kim et al. [24] identified a significant inverse correlation between the FAZ area and BCVA. The mechanism linking a smaller FAZ area with improved postoperative visual outcomes has yet to be elucidated. One potential explanation is that a smaller FAZ may indicate greater neural tissue presence in the MH, enhancing central visual function.

### Limitations

The main limitations of this study are the relatively short follow-up period, which may underestimate late structural and microvascular remodeling that could last up to the first year. Relying on BCVA as the only determinant of functional recovery may overlook other important aspects of visual assessment, such as microperimetry. The absence of a healthy control group or fellow-eye comparison also limits the interpretation of absolute perfusion changes. Lastly, regarding the baseline parameter comparison between both groups, the mean BHD was larger in group B, which may influence the equivalence of the groups.

## Conclusion

This study demonstrated that both free ILM peeling and temporal ILM flap techniques achieved high and comparable anatomical closure rates and sustained visual improvement after idiopathic full-thickness macular hole surgery. Preoperative hole dimensions, particularly BHD, MLD, and HH, emerged as major determinants of structural remodeling and functional recovery, while OCTA revealed substantial microvascular rearrangement, especially within the deep capillary plexus. Overall, postoperative visual function correlated more strongly with restoration of normal retinal architecture, indicating that visual function maintenance relies on both good vascular perfusion and structural restoration.

## Electronic Supplementary Material

Below is the link to the electronic supplementary material.

Supplementary Material 1: SDC1. Surgical technique of modified classic ILM peeling.

Supplementary Material 2: SDC2.Surgical technique of temporal ILM flap.

## Data Availability

All data about the present study are confidential. Access to these data will be granted exclusively to people or entities who meet the criteria for access to confidential data and only upon a written request. All requests should be addressed to the corresponding author Dr. Mohamed H. M. Moustafa.
